# The StarvAnx Study-Comparison Between the Effects of Non-fasting Vs. Fasting Strategy on Surgical Outcomes, Anxiety and Pain in Patients Undergoing Cataract Surgery Under Topical Anesthesia: A Randomized, Crossover, Controlled Trial

**DOI:** 10.3389/fmed.2022.916225

**Published:** 2022-07-13

**Authors:** Gilles Guerrier, Federico Bernabei, Giuseppe Giannaccare, Aldo Vagge, Clémence Bonnet, Christophe Baillard, Dominique Monnet, Pierre-Raphaël Rothschild

**Affiliations:** ^1^Department of Anesthesia and Intensive Care, Cochin Hospital, Assistance Publique-Hôpitaux de Paris (AP-HP), Paris, France; ^2^Université de Paris, Centre de Recherche des Cordeliers, INSERM, Paris, France; ^3^Department of Ophthalmology, Cochin Hospital, Assistance Publique-Hôpitaux de Paris (AP-HP), Paris, France; ^4^Department of Ophthalmology, University Magna Graecia of Catanzaro, Catanzaro, Italy; ^5^University Eye Clinic of Genoa, IRCCS Ospedale Policlinico San Martino, Genoa, Italy

**Keywords:** cataract surgery, pre-operative fasting, anxiety, pain, surgical difficulty

## Abstract

**Background:**

Fasting is usually recommended in patients undergoing cataract surgery under topical anesthesia. However, starving before surgery may increase preoperative anxiety and affect surgical outcomes. It is not known which fasting or non-fasting strategy is best for cataract surgery. The aim of this study was to compare non-fasting and fasting strategy in patients undergoing cataract surgery under topical anesthesia with regard to surgical outcomes, anxiety and pain.

**Methods:**

This randomized, crossover, controlled trial enrolled patients undergoing surgery for bilateral cataract under topical anesthesia at Cochin Hospital (Paris, France), from February to May 2021. Patients were randomly assigned to the non-fasting or fasting group for the first eye surgery and were switched to the other group for the second eye surgery. The primary endpoint was to compare the rate of anesthetist's interventions during surgery. The secondary endpoints included intra-operative complications, duration of surgery, surgeon perception of surgical difficulty, anesthesia-related complications and anxiety and pain level.

**Results:**

one hundred and nine consecutive patients were included, with 60 of them being fasted first and non-fasted for the second eye surgery, while the other 59 were non-fasted first and fasted for the next surgery. The number of patients requiring sedation was significantly lower in the non-fasting group compared with the fasting group [1%; 95%IC (0-3.2) vs. 6%; 95%IC (2.9-8.9), *P* = 0.04]. No anesthesia-related complications were observed. There was no difference in the number of intra-operative complications between the non-fasting and the fasting groups (,respectively, 0 and 1; *P* = 1). Anxiety level and surgical pain were significantly lower in the non-fasting group compared to the fasting group (,respectively, 2.3 ± 2.0 vs. 4.1 ± 2.4, *P* = 0.01 and 0.6 ± 0.6 vs. 2.6 ± 3.4, *P* = 0.003). The mean duration of surgery was significantly shorter in the non-fasting group compared with the fasting group (,respectively, 16.0 ± 5.9 vs. 22.3 ± 6.1 min; *P* = 0.03).

**Conclusion:**

In conclusion pre-operatory non-fasting strategy provides a better patient experience with regards to preoperative anxiety and surgical pain. It allows to reduce operating times and is safe and well-tolerated as regards the anesthetic intervention.

## Introduction

Cataract surgery is among the most commonly performed surgeries in the world (1). Recent innovations in anesthetic and surgical techniques enable to perform most of the procedures under topical anesthesia (2, 3). However, if the patient is particularly stressed at the time of surgery, it is common to inject a sedative before or during the procedure in order to relax the patient and reduce pain and discomfort perception (4, 5). For this reason, patients undergoing cataract surgery under topical anesthesia are often asked to fast before the procedure in order to avoid possible complications due to sedation and analgesia such as regurgitation and aspiration (4). In particular, standard guidelines on preoperative fasting recommend not eating solid foods or drinking clear liquids for 6 and 2 h prior to anesthesia, respectively (6). However, it has been shown that a combination of propofol and ketamine can be safely administered for procedural sedation and analgesia in non-fasted patients without increasing the risk of these complications (7). Furthermore, it has recently been demonstrated that injecting small doses of sedative, including propofol, in non-fasted patients, does not increase the risk of complications (8). For these reasons, preoperative fasting in this patient population remains still controversial (5).

It is known that hunger and thirst can exacerbate anxiety and adversely affect patient comfort and satisfaction in different surgical populations (9, 10). Moreover, it has been showed that fasting patients have a greater response to pain and therefore need for additional analgesics (11).

To date, the impact of fasting on surgical performance has not been documented in cataract surgery. It can be assumed that fasting induces a greater state of anxiety, which in turn results in poor patient cooperation, increasing the risk of intraoperative complications and reducing patient satisfaction. The aim of this randomized, crossover, control trial was to compare the rate of anesthetist's interventions, surgical complications and different patients' satisfaction parameters including anxiety level and intraoperative pain perception between fasted and non-fasted patients undergoing phacoemulsification surgery for age-related cataract under topical anesthesia.

## Materials and Methods

### Design and Patients

This randomized, crossover, controlled trial was conducted between February 2021 and May 2021 at the Ophthalmology Service, OphtalmoPôle de Paris of the Cochin Hospital (Paris, France). The study was performed in accordance with the principles of the Declaration of Helsinki and was approved by the University's Institutional Review Board (IRB 00010259-2020-004). The trial was registered prior to patient enrollment at ClinicalTrials.gov (NCT04769856, Date of registration: 25/02/2021). Written informed consent was obtained from all study subjects. Patients with age-related cataract scheduled for bilateral surgery under topical anesthesia were screened to be enrolled in the study. Exclusion criteria were the presence of other ocular co-morbidities such as exfoliation syndrome, uveitis, myopia with axial length > 26 mm, hyperopia with axial length <21 mm, posterior synechiae, phacodonesis, diabetes, chronic cough, hearing impairment, psychiatric disorders, dementia, strabismus or poor fixation due to nystagmus. In addition, patients with complicated cataract and hard nuclear cataracts (nuclear opalescence scores 5 or greater on Lens Opacities Classification System-III system) were excluded from the study (12).

### Randomization Process and Surgical Procedure

The included patients were randomly assigned to the fasting or the non-fasting group for the first surgery, and then switched to the other group for the second surgery 2 weeks later. The randomization process was carried out the day before surgery *via* a computer-generated, interactive web-response system (Cleanweb^®^, Telemedecine technologies S.A.S, Boulogne-Billancourt, France) and patients were informed by SMS.

The instruction of preoperative fasting, in case they were included in the fasting group, was explained to all patients on the day of the consultation with the anesthetist when written informed consent was obtained. On the day of surgery, patients in the fasting group were prohibited from taking solids and liquids starting 6 and 2 h before surgery, respectively. Patients in the non-fasting group were advised not to make any changes to their daily routines, including eating and drinking before admission.

The day of the surgical procedure surgeons, anesthetists and all the nursing staff were blinded to randomization group.

On the day of surgery, all patients with chronic diseases received their usual medications, including heart and antihypertensive medications. No sedative premedication was given to patients prior to surgery. Blood pressure, heart rate, and plethysmography oxygen saturation were monitored during the procedure. A 22 G intravenous cannula was inserted before surgical procedure. No additional fluids were given intravenously before the operation in either group. Sedation was administered by the attending nurse when deemed necessary by the surgeon, consisting of a low dose of propofol (0.25 mg/kg) repeated once if required with nasal oxygen delivered at 2 L/min.

Cataract surgery was performed at one eye of each patient under topical anesthesia by an experienced surgeon (CB, PRR, DM). Topical anesthesia consisted in administration of oxybuprocaine 0.5% drops into the conjunctival sac 3 times in the 15 min preceding surgery. The primary steps of the surgery were a self-sealing temporal limbal 2.2 mm incision, subsequently capsulorhexis, hydrodissection and phacoemulsification were performed. Finally, a foldable intraocular lens was inserted in the capsular bag. Intracameral 1 mg cefuroxime injection was administered at the end of surgery. Postoperative topical therapy consisted of fluoroquinolone eye drops for 1 week and dexamethasone eye drops for 1 month.

### Data Collection

The primary endpoint was to compare the rate of anesthetist's interventions. Secondary endpoints included intra-operative complications, in particular, posterior capsule rupture, duration of surgery, surgeon perception of surgical difficulty, anesthesia related complications, patients' anxiety and pain level and perception of hunger and thirst. The duration of surgery was measured as the time from insertion to removal of the speculum. The surgeon judged the difficulty of the surgery, based on patient compliance and cooperation, on a scale of 0 to 5 (where 0 is full cooperation and no difficulty with the surgery, and 5 is a situation where surgery was impossible due to an uncooperative patient, and sedation had to be instituted). Primary and secondary outcomes were blindly assessed by a second research assistant (FB). Pre-operative anxiety level and intra-operative pain were measured by the anesthetist using the 0-10 verbal rating scale (VRS), respectively, before surgical procedure and during the phacoemulsification of the cataract (13). Furthermore, before starting the surgery the patient was asked if he perceived the sensation of hunger and thirst. Pre-operative waiting time, i.e., the time from the moment the patient was ready for surgery until transfer to the operating room, was also recorded. Furthermore, the duration of fasting at the time of the intervention was recorded in both groups. Compliance with the allocated group was assessed by a research assistant the morning of surgery at admission.

### Data Analysis

The SAS 9.4 statistical software (Copyright© 2016 by SAS Institute Inc., Cary, NC, United States) was used for data analysis. Continuous and ordinal data were presented as mean ± standard deviation, while categorical data were represented by number, percentage and 95% confidence interval.

The rate of anesthetist's intervention was estimated at 18% among fasted patients in our center. A sample size of 60 patients per arm was needed to detect a 50% reduction in the number of anesthetist's intervention, at the 0.05 significance level with 0.8 power. Unless otherwise specified, categorical variables were compared by a Chi-square test or Fisher's exact test as appropriate, and continuous variables were compared by a Student's *t*-test or Wilcoxon-Mann-Whitney test as appropriate. The Pearson's correlation method was used to investigate possible correlations between anxiety level and fasting times. All calculations were performed using SAS statistical software version 9.4 (SAS Institute).

## Results

Overall, 149 patients were recruited for eligibility. Of these, 24 did not meet the inclusion criteria and 2 declined to participate. Finally, a total of 123 consecutive patients were enrolled in the study and randomly assigned to one of the 2 preoperative fasting regimens for the first eye cataract surgery and switched to the other group for the second eye surgery. In particular, 62 were allocated to the non-fasting group for the first eye and the fasting group for the second eye, and 61 were allocated to the fasting group for the first eye and to the non-fasting group for the second eye. On the day of surgery, 3 patients in the non-fasting group and 1 in the fasting group did not comply with the protocol and were excluded from the study. Finally, data of 119 patients were used for the analysis.

Table 1 shows baseline characteristics of patients undergoing cataract surgery including age, sex distribution, ASA (American Society of Anesthesiologists Classification) score and the number of hypertensive patients.

**Table 1:** Baseline characteristics of patients undergoing cataract surgery.

**Characteristics**	**Mean ± SD or *n* (%)**
Age (yr)	68.6 ± 10.7
Sex (Female)	86 (72%)
ASA score	I/II 78 (66%) III/IV 41 (34%)
Hypertension	75 (63%)

*ASA, American Society of Anesthesiologists Classification*.

The number of patients who required sedation during the procedure was significantly lower in the non-fasting group than in the fasting group [,respectively, 1 (1 %) (0-3.2) vs. 5 (6 %) (2.9-8.9), *P* = 0.04]. One intraoperative complication (posterior capsule rupture) was observed in the fasting group, but the difference was not significant compared to the non-fasting group (*P* = 1). The mean duration of surgery was significantly shorter in the non-fasting group compared with the fasting group (,respectively, 16.0 ± 5.9 vs. 22.3 ± 6.1 min; *P* = 0.03). Surgeons reported higher difficulty to perform the procedure in fasting than non-fasting patients (,respectively, 1.9 ± 2.0 vs. 0.4 ± 0.7; *P* = 0.02). No anesthesia-related complications were reported. Patients in the non-fasting group exhibited lower levels of preoperative anxiety and surgical pain than those in the non-fasting group (,respectively, 2.3 ± 2.0 vs. 4.1 ± 2.4; *P* = 0.01 and 0.6 ± 0.6 vs. 2.6 ± 3.4; *P* = 0.003). Before starting surgery, patients in the fasting group reported perception of thirst more often than patients in the non-fasting group: 98 [82 %; 95% CI (74-99)] vs. 24 [20 %; 95%CI (18-31); *P* < 0.001]. Similarly, patients in the fasting group reported perception of hunger more often than those in the non-fasting group, respectively, 46 [38%; 95%CI (29-45)] vs. 22 [18%; 95%CI (15-29); *P* = 0.02]. There were no significantly differences in pre-operative waiting time between the non-fasting and fasting group (,respectively 63 ± 45 and 61 ± 48 min, *P* > 0.05). On admission, the mean duration of fasting for solids and liquids in the fasting group were 724 ± 292 and 221 ± 98 min, respectively. The non-fasting group consumed solid 83 ± 32 min and liquids 40 ± 63 min before surgery (all *P* < 0.001).

Table 2 shows discomfort, anxiety, and pain perception in the 2 groups.

**Table 2:** Discomfort, anxiety, and pain perception in the two groups.

	**Non - Fasting *n* (%) [95%IC] or Mean ± SD**	**Fasting *n* (%) [95%IC] or Mean ± SD**	***P*** **value (chi-squared test)**
**Perception of hunger before starting surgery**			
*N* = 119	22 (18) [15–29]	46 (38) [29-45]	0.02
First eye	10 (17) [12–26]	25 (42) [30-58]	0.01
Second eye	12 (20) [17–28]	21 (35) [24-49]	0.03
**Perception of thirst before starting surgery**			
*N* = 120	24 (20) [18–31]	98 (82) [74-99]	< 0.001
First eye	12 (20) [10–29]	51 (85) [71-99]	< 0.001
Second eye	12 (20) [8–21]	47 (78) [70-99]	< 0.001
**Pre-operative anxiety level (VRS)**			
*N* = 119	2.3 ± 2.0	4.1 ± 2.4	0.01
First eye	2.2 ± 1.8	4.9 ± 2.1	0.01
Second eye	2.4 ± 2.3	3.9 ± 2.9	0.02
**Surgical pain (VRS)**			
*N* = 119	0.6 ± 0.6	2.6 ± 3.4	0.003
First eye	0.5 ± 0.9	2.8 ± 3.0	0.002
Second eye	0.7 ± 0.8	2.5 ± 2.4	0.01

*VRS: 0-10 Verbal Rating Scale*.

Table 3 shows fasting durations and pre-operative waiting times in the 2 groups.

**Table 3:** Fasting durations and pre-operative waiting times in the two groups.

	**Non - Fasting Mean ± SD**	**Fasting** **Mean ± SD**	***P*** **value** **(*t*- test)**
**Fasting duration on admission (min)**			
***N*** **= 119**			
Solids	83 ± 32	724 ± 292	< 0.001
Liquids	40 ± 63	221 ± 98	< 0.001
First eye			
Solids	95 ± 42	774 ± 411	< 0.001
Liquids	45 ± 61	252 ± 58	< 0.001
**Second eye**			
Solids	71 ± 39	624 ± 390	< 0.001
Liquids	38 ± 53	190 ± 118	< 0.001
**Pre-operative waiting time (min)**			
*N* = 119	63 ± 45	61 ± 48	0.23
First eye	93 ± 49	89 ± 28	0.42
Second eye	50 ± 37	54 ± 33	0.11
**Fasting duration on starting surgery (min)**			
***N*** **= 119**			
Solids	165 ± 42	781 ± 154	< 0.001
Liquids	164 ± 61	282 ± 77	< 0.001
First eye			
Solids	188 ± 44	863 ± 119	< 0.001
Liquids	138 ± 52	341 ± 68	< 0.001
**Second eye**			
Solids	213 ± 50	678 ± 299	< 0.001
Liquids	180 ± 73	244 ± 102	< 0.001

In the fasting group, there was a correlation between anxiety level and fasting time for liquids in (r = 0.83; *P* < 0.001), while no correlation was found between anxiety level and fasting time for solids (r = 0.12; *P* = 0.20). Similarly, there was no correlation between pain level and fasting time for both liquids and solids (,respectively, r = 0.21; *P* = 0.3 and r = 0.15; *P* = 0.1).

**Figure 1:**
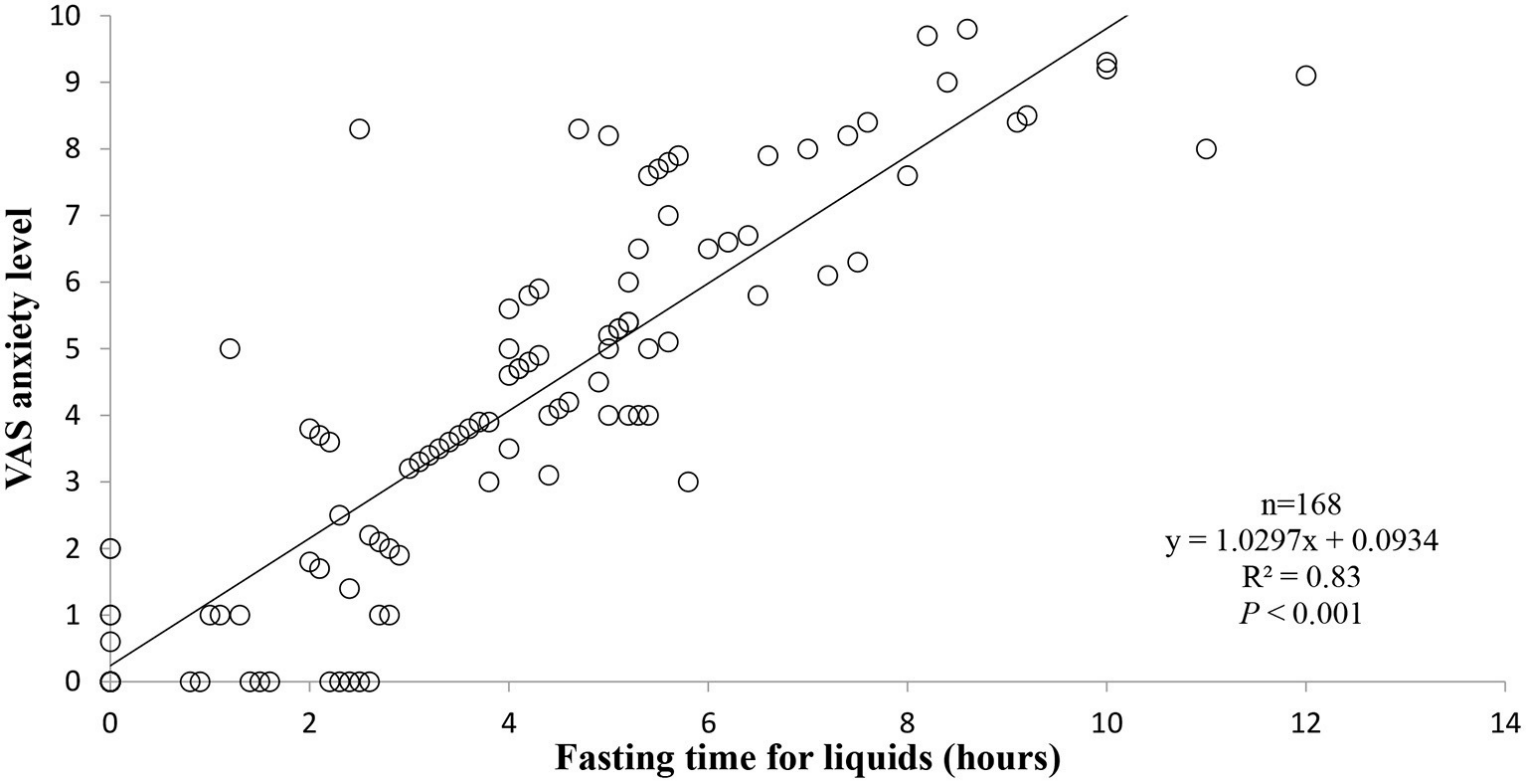
Correlations between anxiety level and fasting time for liquids.

Figure 1 shows the correlations between anxiety level and fasting time for liquids.

## Discussion

This randomized, crossover, controlled trial showed that non-fasting patients required significantly less sedations during cataract surgery under topical anesthesia. Indeed, sedation was required in < 1% of non-fasting patients compared with 6% in fasting patients and no anesthesia-related adverse event was reported. These findings confirm those already reported in the literature, namely that no cases of aspiration pneumonia have been recorded after the use of procedural sedation in non-fasting patients, including in cataract surgery (6, 8). Along with these results we observed that patients in the non-fasting group had lower levels of anxiety and intraoperative pain than those in the fasting group. Previous studies performed in different surgical populations showed that the perception of pain is positively correlated with preoperative anxiety (14, 15). Additionally, preoperative fasting has been shown to increase anxiety levels (9, 16, 17). For this reason, it can be assumed that non-fasting patients enter the operating room more relaxed and experience a lower perception of pain related to the surgical procedure. This could contribute to reduce patient discomfort during surgery with less unexpected movements and agitation as well as making the surgery easier for the surgeon. Moreover, elevation of intraocular pressure is one major factor increasing surgical difficulty in cataract surgery. Anxiety and pain can lead patients to Valsava-like maneuvers, resulting in increased central venous pressure and subsequently intraocular pressure that alters intraoperative conditions.

Of relevance, we found a significant correlation between anxiety and fasting times. A previous study reported greater intraoperative pain perception and higher anxiety levels in fasted patients when the surgery was performed in the afternoon than in the morning (18). This result may have been due to the fact that patients fasted longer than those operated in the morning. In addition, it has been reported that preoperative anxiety is associated with the level of pain experienced during routine cataract surgery (7). It is plausible that anxiety or pain reduce capacity of understanding information and following instructions during surgery. As the patient remains conscious during the procedure and fully aware of the various stages, anxiety and pain may be exacerbated by the perception of thirst and hunger.

Regarding intra-operative complications, one capsular rupture was observed in the fasting group, and no complication was observed in the non-fasting group. Since this difference was not significant, it is therefore not possible to conclude that the non-fasting regimen results in a lower incidence of complications. This result deserves to be confirmed by studies with a very high number of patients included as it is known that when surgery is performed by experienced surgeons the complication rate is relatively low, in particular, < 1% (19).

Interestingly, the surgical procedure was found to be faster in the non-fasting group. In addition, surgeons reported having less difficulty performing surgery in this group. The present study shows further evidence that patients should be allowed to consume solids or liquids prior to cataract surgery performed under topical anesthesia, regardless of intravenous sedation requirement. Additionally, fasting times are commonly extended beyond recommended times, especially in medical teaching facilities, where delays in the operating room often occur with a major effect on patient flow and subsequent postponement, cancellation, and rescheduling of procedures. The non-fasting strategy can improve management efficiency to mitigate subsequent adverse surgical outcomes and improve overall satisfaction in cataract surgery centers.

The strength of this study is the crossover design that prevented biases such as age, gender or past medical history of patients, including previous surgical procedures. We identified some limitations for the present study that deserve mentioning. First, given the low incidence of posterior capsule rupture when surgery is performed by an experienced surgeon, the sample size was likely small to show a difference in the complication rate between the two groups. Further studies with a larger number of patients are desirable to investigate this issue. Second, the present study was carried out in a high-volume, well-equipped, and specialized teaching surgical center. The validity of the results of this study needs to be demonstrated in other settings and other populations, including diabetic patients. Currently, there are no internationally accepted guidelines specific to the perioperative management of diabetic patients undergoing cataract surgery (11). Third, although recognized as a valid indicator and widely used in clinical trials, VRS may not reliably reflect anxiety as it is a highly subjective assessment method. Four, we have not carried out a cost-efficiency analysis which would be useful in determining which of the two strategies is the best. This issue should be addressed in future studies. Five, chronic treatment compliance was not assessed in this study. This would be interesting to investigate as in our experience we observed that fasting patients have a tendency to disregard adherence to treatment on the day of surgery. Finally, the time between admission to the surgical procedure was not recorded. However, it is unlikely that there was a difference between the two groups in patient preparation time.

In conclusion, the pre-operatory non-fasting strategy in patients undergoing cataract surgery under topical anesthesia reduces the need for sedation and provides a better patient experience with regards to anxiety and pain. Furthermore, the surgeon activity is facilitated by the better disposition of the patient, experiencing less difficulty in carrying out the procedure. Finally, this strategy allows to reduce operating times and is safe and well-tolerated as regards the anesthetic intervention.

## Data Availability Statement

The raw data supporting the conclusions of this article will be made available by the authors, without undue reservation.

## Ethics Statement

The studies involving human participants were reviewed and approved by Sorbonne University's Institutional Review Board (IRB 00010259 - 2020 - 004). The patients/participants provided their written informed consent to participate in this study.

## Author Contributions

GGu designed the study. GGu, DM, and P-RR supervised the work. FB, AV, and GGi analyzed data. GGu, GGi, FB, CBo, CBa, DM, AV, and P-RR wrote the paper, read, and approved the final manuscript. All authors contributed to the article and approved the submitted version.

## Conflict of Interest

The authors declare that the research was conducted in the absence of any commercial or financial relationships that could be construed as a potential conflict of interest.

## Publisher's Note

All claims expressed in this article are solely those of the authors and do not necessarily represent those of their affiliated organizations, or those of the publisher, the editors and the reviewers. Any product that may be evaluated in this article, or claim that may be made by its manufacturer, is not guaranteed or endorsed by the publisher.
