# Supplementary figures and images for: The StarvAnx Study-Comparison Between the Effects of Non-fasting Vs. Fasting Strategy on Surgical Outcomes, Anxiety and Pain in Patients Undergoing Cataract Surgery Under Topical Anesthesia: A Randomized, Crossover, Controlled Trial
Source: Front Med (Lausanne). 2022 Jul 13;9:916225. doi: 10.3389/fmed.2022.916225 (PMC9326043; doi:10.3389/fmed.2022.916225)

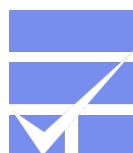

# CONSORT

TRANSPARENT REPORTING of TRIALS

## CONSORT Flow Diagram

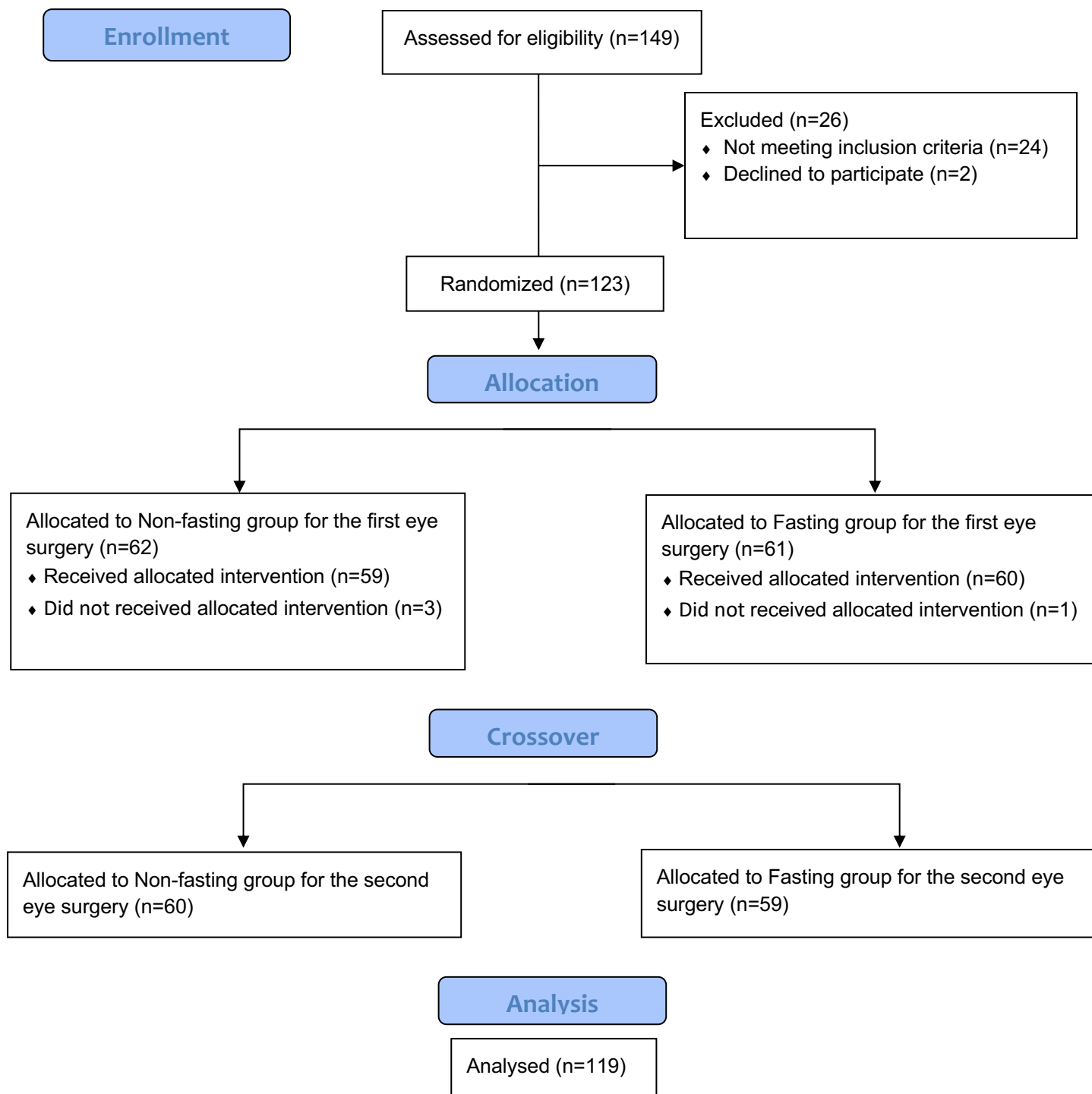

Supplement: Supplementary file 1 [file Data_Sheet_1.PDF]
